# Wide cross-reactivity between *Anopheles gambiae *and *Anopheles funestus *SG6 salivary proteins supports exploitation of gSG6 as a marker of human exposure to major malaria vectors in tropical Africa

**DOI:** 10.1186/1475-2875-10-206

**Published:** 2011-07-27

**Authors:** Cinzia Rizzo, Raffaele Ronca, Gabriella Fiorentino, Valentina D Mangano, Sodiomon B Sirima, Issa Nèbiè, Vincenzo Petrarca, David Modiano, Bruno Arcà

**Affiliations:** 1 Department of Public Health and Infectious Diseases, "Sapienza" University, P.le Aldo Moro 5, 00185 Rome, Italy; 2 Department of Structural and Functional Biology, "Federico II" University, Monte S. Angelo Campus, Via Cinthia 4, 80126 Naples, Italy; 3 Centre National de Recherche et de Formation sur le Paludisme, 01 BP 2208 Ouagadougou 01, Burkina Faso; 4 Department of Biology and Biotechnology "Charles Darwin", Sapienza University, P.le Aldo Moro 5, 00185 Rome, Italy; 5 Istituto Pasteur, Fondazione Cenci Bolognetti, "Sapienza" University, Italy

## Abstract

**Background:**

The *Anopheles gambiae *gSG6 is an anopheline-specific salivary protein which helps female mosquitoes to efficiently feed on blood. Besides its role in haematophagy, gSG6 is immunogenic and elicits in exposed individuals an IgG response, which may be used as indicator of exposure to the main African malaria vector *A. gambiae*. However, malaria transmission in tropical Africa is sustained by three main vectors (*A. gambiae*, *Anopheles arabiensis *and *Anopheles funestus*) and a general marker, reflecting exposure to at least these three species, would be especially valuable. The SG6 protein is highly conserved within the *A. gambiae *species complex whereas the *A. funestus *homologue, fSG6, is more divergent (80% identity with gSG6). The aim of this study was to evaluate cross-reactivity of human sera to gSG6 and fSG6.

**Methods:**

The *A. funestus *SG6 protein was expressed/purified and the humoral response to gSG6, fSG6 and a combination of the two antigens was compared in a population from a malaria hyperendemic area of Burkina Faso where both vectors were present, although with a large *A. gambiae *prevalence (>75%). Sera collected at the beginning and at the end of the high transmission/rainy season, as well as during the following low transmission/dry season, were analysed.

**Results:**

According to previous observations, both anti-SG6 IgG level and prevalence decreased during the low transmission/dry season and showed a typical age-dependent pattern. No significant difference in the response to the two antigens was found, although their combined use yielded in most cases higher IgG level.

**Conclusions:**

Comparative analysis of gSG6 and fSG6 immunogenicity to humans suggests the occurrence of a wide cross-reactivity, even though the two proteins carry species-specific epitopes. This study supports the use of gSG6 as reliable indicator of exposure to the three main African malaria vectors, a marker which may be useful to monitor malaria transmission and evaluate vector control measures, especially in conditions of low malaria transmission and/or reduced vector density. The *Anopheles stephensi *SG6 protein also shares 80% identity with gSG6, suggesting the attractive possibility that the *A. gambiae *protein may also be useful to assess human exposure to several Asian malaria vectors.

## Background

After more than a century from the discovery of the role of *Anopheles *mosquitoes in the transmission of *Plasmodium *parasites, malaria is still one of the leading causes of human morbidity and mortality. Currently, the malaria toll is especially high among young children in sub-Saharan Africa, where transmission of the most deadly malaria parasite, *Plasmodium falciparum*, is mainly accomplished by two members of the *Anopheles gambiae *species complex (i.e. *A. gambiae *and *Anopheles arabiensis*, subgenus *Cellia*, Pyretophorus Series) and by *Anopheles funestus *(subgenus *Cellia*, Myzomyia Series) [1]. Proper evaluation of malaria transmission intensity, of seasonal and temporal variation of vector density and of the efficacy of anti-parasite and anti-vector control measures play crucial roles in the framework of anti-malaria strategies.

Assessment of malaria transmission intensity is currently based on both parasitological and entomological measures and a key parameter is the entomological inoculation rate (EIR), which accounts for human exposure to parasite-carrying mosquitoes. However, entomological measurements are not only expensive and labor-intensive but, sometime, also difficult or impossible to apply: for example in conditions of low transmission intensity and/or low mosquito density, or for logistic restrictions. Therefore, additional and/or alternative methods to evaluate *Anopheles *density and human exposure to malaria vectors would be extremely valuable allowing for epidemiological studies also in settings where classical entomological methods are of problematic use. During blood feeding, mosquitoes inject into their hosts a complex mixture of salivary components whose main role is to facilitate haematophagy by counteracting the haemostatic, inflammatory and immune responses of vertebrates [2,3]. These salivary components also elicit into hosts an immune response with production of anti-saliva antibodies. For example, at least 10-15 protein bands recognized by human IgG can be detected by western blot using *A. gambiae *salivary gland protein extracts and sera from exposed individuals from a malaria hyperendemic area (B.A., unpublished observations). Several reports support the concept that measurement of this antibody response to saliva may represent an indicator of human exposure to *Anopheles *bites and malaria risk, as well as a tool to evaluate efficacy of insecticide-treated nets (ITNs) [4-8]. Moreover, the identification of *Anopheles*-specific proteins, i.e. not found in other mosquitoes or blood feeding arthropods, offers the opportunity to use as markers genus-specific recombinant salivary antigens instead of saliva [2,9]. This enables for a significant improvement of the methodology increasing both the accuracy and the specificity by overcoming the need of obtaining large amount of saliva and potential problems of reproducibility and cross-reactivity.

Conveniently, vector's salivary antigens could be used in parallel to *Plasmodium *antigens to assess, by serological determination of antibody levels, both exposure of humans to *Anopheles *mosquitoes and malaria transmission intensity [10-14]. This immune response to salivary antigens would represent a direct measure of intensity of mosquito biting activity on humans, both at the population and at individual level, and could provide a few additional advantages. First, it may allow to assess *Anopheles *exposure in children, which is presently unworkable for ethical reasons (the method currently in use is based on human landing catches on adult volunteers). Second, it would be very helpful to evaluate the impact of anti-vector control measures on exposure of humans to *Anopheles *bites. Third, it would be a tool especially needed for epidemiological assessments in areas of low malaria transmission, which are currently increasing as a consequence of the decline of the malaria burden in several areas of sub-Saharan Africa [15]. Finally, it might be the appropriate tool to verify if, and eventually to what extent, the mosquito biting activity is heterogeneously distributed within a population. Indeed, according to the so-called heterogeneous biting model, mosquito biting may be unequally distributed, with few people receiving most of the mosquito bites (i.e. 20-30% of the population getting 70-80% of the bites). Heterogeneous biting has broad implications for malaria epidemiology and control and, as recently suggested, may provide a plausible explanation for inconsistencies related to malaria transmission dynamics and modelling [16].

Toward the development of serological markers of exposure to malaria vectors, attention was focused on gSG6 (**g**ambiae **S**alivary **G**land protein **6**), a small protein initially identified in *A. gambiae*, where it is specifically expressed in the salivary glands of adult female mosquitoes [17]. Its specific function awaits full clarification; however, gSG6 must play some relevant role in haematophagy since its depletion by RNAi increases probing time and affects blood feeding ability [18]. Afterwards, members of the SG6 protein family have been identified in the salivary transcriptomes of additional anopheline mosquitoes, but in no other living organisms, pointing to its genus-specificity and blood feeding role. Among the few anophelines analysed so far the SG6 protein is present in species belonging to the subgenus *Cellia *(*A. gambiae *species complex, *A. funestus*, *Anopheles stephensi*) and in *Anopheles freeborni *(a member of the subgenus *Anopheles*), but it is notably absent in *Anopheles darlingi*, a member of the subgenus *Nyssorhynchus *and vector of malaria in Central and South America [18]. This observation suggests that SG6 family members may be widely distributed among the main African and Asian malaria vectors, but most likely absent in South American ones.

Given the anopheline-specificity and previous indications of the immunogenicity to humans of gSG6-based peptides [19], the *A. gambiae *gSG6 protein was expressed in recombinant form and the anti-gSG6 IgG response was analysed in a population from a malaria hyperendemic area of Burkina Faso. This study provided experimental evidence that gSG6 may be a good candidate as serological marker of human exposure to *A. gambiae *[20], although full validation in different epidemiological settings (i.e. low transmission conditions, macro-geographic scale) is needed. Moreover, since malaria is transmitted by multiple and often sympatric vectors, an ideal salivary marker should allow to estimate exposure to all the major vector species in the study area. The *A. gambiae *gSG6 protein is highly conserved among members of the *A. gambiae *species complex (99% identity with the *A. arabiensis *homologue, aSG6), whereas it is more distantly related (80%, 70/87 residues) to the *A. funestus *protein (fSG6). It is likely that a certain degree of cross-reactivity to the two protein exists, but the extent of the overlap of the human IgG response to gSG6 and fSG6 proteins is unknown. Some indications in this direction have been obtained using the 23 aa long gSG6-P1 peptide, which encompasses the gSG6 N-terminal region [21]. However, this peptide is less sensitive in comparison to the whole protein (approx 5-fold) and, therefore, it would be important to experimentally validate the efficacy of using the antibody response to the gSG6 protein as marker of exposure to the three main malaria vectors in tropical Africa. The aim of this study was to evaluate cross-reactivity of human sera from exposed individuals to the gSG6 and fSG6 proteins. To this purpose the *A. funestus *fSG6 was expressed in recombinant form and the IgG response to fSG6, gSG6 and to an equimolar mixture of the two proteins was compared by ELISA using sera of individuals from a malaria hyperendemic area of Burkina Faso.

## Methods

### Study area and entomological observations

Surveys were carried out in the village of Barkoumbilen (~35 km NE of Ouagadougou, Burkina Faso), a rural settlement inhabited by the two ethnic groups Mossi and Rimaibé. The area was characterized by intense *P. falciparum *transmission, mostly linked to the rainy season (from June to October), with entomological inoculation rates >100/person/year. Malaria prevalence was very high, *P. falciparum *representing about 95% of malaria infections, and infection rates ranged, during the high transmission season, from 60% to 90% according to age group. Lower prevalences, ranging between 40% and 80%, were observed during the dry low transmission season. The study protocol was approved by the Technical Committee of the Centre National de Lutte contre le Paludisme of the Ministry of Health of Burkina Faso. Oral informed consent for multiple immuno-parasitological, clinical and entomological surveys was obtained from the Mossi-Rimaibè community living in the village of Barkoumbilen. A total of 335 sera collected from individuals of the Mossi ethnic group in 1994 at the beginning (August '94) and at the end (October '94) of the high transmission/rainy season, as well as in the following low transmission/dry season (March '95) were analysed in this study. Sera from 48 Roman citizens (1-56 years old) who were referred to a city hospital for routine blood testing were used as a control. Additional information on samples size and average age for each survey can be found in the legends to Figures. Entomological measures were based on indoor pyrethrum spray catches carried out monthly between August and November '94 and in March '95 (12 catches/month). A total of 1,653 female *Anopheles *mosquitoes were identified: among these 1,479 were members of the *A. gambiae *species complex (*A. gambiae *or *A. arabiensis*) and 174 were *A. funestus*. Additional details on the study site and on entomological and parasitological aspects have been previously reported and can be found elsewhere [22-24].

### Protein expression and purification

The *A. funestus *SG6 protein (fSG6) was expressed as N-terminal His-tagged recombinant protein in the *E. coli *vector pET28b(+) (Novagen). Briefly, the region encoding the mature fSG6 protein was PCR amplified using as template genomic DNA extracted from a single mosquito collected in 2008 in the Bobo-Dioulasso area, Burkina Faso. Amplification was performed using the *Pfx *DNA polymerase (Invitrogen) and the oligonucleotide primers G6fu-Nde (5'- GTCTCATATGGAAAAGGTTTGGGTCGATCG-3'OH) and G6fu-Eco (5'- GTCTGAATTCTCACTGTTCCAGGAAGGGTTTG -3'OH). Directional cloning in the *Nde*I/*Eco*RI-digested pET28b vector yielded the pET-fSG6 expression vector, that was sequenced and then introduced into competent BL21(DE3)RIL *E. coli *cells (Stratagene). Expression and purification was essentially performed as previously reported for the *A. gambiae *gSG6 protein [20] with few modifications. After overnight growth (37°C, LB medium) 5 ml of the saturated culture were transferred into 400 ml of LB medium and grown up to 0.8 OD_600 _before starting induction by IPTG (0.1 mM). After 4 hours cells were harvested and the pellet resuspended in 20 ml of 50 mM Tris-HCl pH 8.0, 50 mg/ml lysozyme and sonicated. Inclusion bodies (IB) were collected by centrifugation (15,000 g, 20 min, 4°C), resuspended in extraction buffer (50 mM Tris-HCl pH 8.0, 2 M Urea, 5 mM EDTA, 1% Triton-X100), washed twice (50 mM Tris-HCl pH 8.0, 2 M Urea) and centrifuged as above. Proteins from IB were solubilized by gentle shaking over-night (5 ml of 20 mM Na_2_HPO_4_, 6 M Guanidine-HCl, 0.5 M NaCl, 5 mM Imidazole, pH 8.0), centrifuged (20000 g, 30 min, 4°C) and subjected to affinity chromatography under denaturing conditions (HisTrap, GE Healthcare) according to manufacturer's instructions. Fractions containing the His-tagged fSG6 were identified by SDS-PAGE and pooled. Refolding of this fSG6-enriched fraction was carried out by rapid dilution in 20 volumes of refolding buffer (100 mM Tris-HCl pH 8.0, 500 mM L-Arg, 300 mM NaCl, 5 mM L-Glutathione reduced, 0.5 mM L-Glutathione oxidized) and left for 24 h at 4°C with low stirring. After centrifugation (20,000 g, 30 min, 4°C) the refolded proteins were concentrated by centrifugation in Amicon^® ^Ultra (5 kDa MWCO, Millipore), dialyzed against 20 mM Tris-HCl pH 8.0 and further purified by anion exchange chromatography (HiTrapQ, GE Healthcare). Elution was carried out with a linear gradient 0 - 0.5 M NaCl in 18 column volumes. Fractions containing the fSG6 recombinant protein were identified by SDS-PAGE, pooled, concentrated and dyalized against 20 mM Tris-HCl pH 8.0, 150 mM NaCl. Concentration of the purified protein was estimated by the Bradford Protein Assay (Bio-Rad Laboratories). The yield of purified protein was of approximately 6 mg/l, however this is most likely susceptible to improvements since optimization of the procedure for the *A. gambiae *homologue allowed for higher recovery (~9-12 mg/l) as previously reported [20].

### Enzyme-Linked ImmunoSorbent Assay SG6

ELISA was performed according to standard procedures. Maxisorp 96-well plates (Nunc M9410) were coated overnight at 4°C with 50 μl of the *A. gambiae *gSG6 (10 μg/ml), or the *A. funestus *fSG6 (10 μg/ml), or with an equimolar mixture of the two protein (10 μg/ml total) in coating buffer (15 mM Na_2_CO_3_, 35 mM NaHCO_3_, 3 mM NaN_3_, pH 9.6). After washing (four times) wells were blocked (3 hrs, RT) in 150 μl of 1% w/v skimmed dry milk in PBST (PBS Sigma P4417+0.05% Tween 20), washed again and incubated overnight at 4°C with 50 μl of serum (1:100) in blocking buffer. Sera were analysed in duplicate with each antigen and once without antigen (coating buffer only). Each plate included a two-fold dilution series (1:40 to 1:2560 final dilutions) of a standard African hyperimmune sera pool. After washing plates were incubated (3 hrs, RT) with 100 μl of polyclonal rabbit anti-human IgG/HRP antibody (Dako P0214, 1:5000 in blocking buffer). After washing as above the colorimetric development was carried out (15 min, RT in the dark) with 100 μl of o-phenylenediamine dihydrochloride (OPD, Sigma P8287). The reaction was terminated adding 25 μl of 2 M H_2_SO_4 _and the OD_492 _was determined using a microplate reader (BioTek Synergy HT). IgG levels were expressed as final OD calculated for each serum as the mean OD value with antigen minus the OD value without antigen. OD values were normalized using the titration curve as previously described [25]. The normalized ODs were calculated using the Excel software (Microsoft) with a three variable sigmoid model and the Solver add-in application.

### Data analysis

Sera whose duplicates showed a coefficient of variation (CV) >20% were not included into the analysis. The mean optical density (OD) of unexposed controls plus 3 standard deviations (SD) was used as cut-off value for seropositivity. Cut-off values were 0.140 for gSG6, 0.160 for fSG6 and 0.132 for the two antigens combined. Individuals showing OD values above the cut-off level for seropositivity were classified as responders. Multiple comparisons were performed by the Kruskal-Wallis test or, for matched groups, by the Friedman's test. Mann-Whitney U test was used to compare IgG levels among responders of two independent groups. The Wilcoxon matched-pairs test was used for comparison of two paired groups. Frequencies were compared by the chi-square test. All statistical analysis was performed using GraphPad Prism 5.0^® ^statistical software (GraphPad Software Inc., La Jolla, CA).

## Results and discussion

### Expression and purification of the *A. funestus *SG6 protein

**Figure 1:**
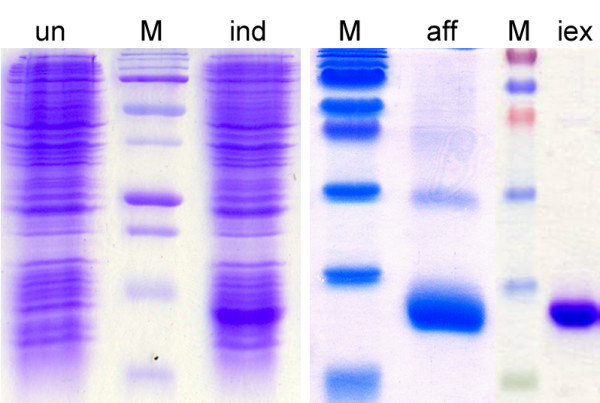
**Expression and purification of the *A. funestus *fSG6**. The recombinant fSG6 was expressed in *E. coli *Bl21(DE3)RIL cells. SDS-PAGE analysis of protein fractions from uninduced (un) and induced (ind) cells are shown on the left panel. Protein fractions obtained after His-Trap affinity chromatography (aff) were pooled, further purified by ion exchange chromatography (iex) and analysed by SDS-PAGE (right panels). M, molecular weight markers.

Taking advantage of the intron-less nature of SG6 family members [18] the region encoding the mature fSG6 protein was amplified by PCR using *A. funestus *genomic DNA as template. Sequence analysis showed a single non-synonymous substitution (G to C) which results in an amino acid replacement (K to N) in position 78 of the mature polypeptide in comparison to the previously reported sequence [GenBank:DQ910319]. This substitution was interpreted as a naturally occurring polymorphism, rather than a PCR-introduced error, both for the proofreading activity of the thermostable DNA polymerase used for PCR amplification, and because the ESTs database includes several entries carrying the same non-synonymous SNP. The fSG6 protein was expressed as N-terminal His-tagged recombinant protein in *Escherichia coli *and purified from inclusion bodies by affinity and ion-exchange chromatography (Figure 1) according to the procedure previously optimized for the *A. gambiae *homologous protein [20].

### Study area and entomological data

**Figure 2:**
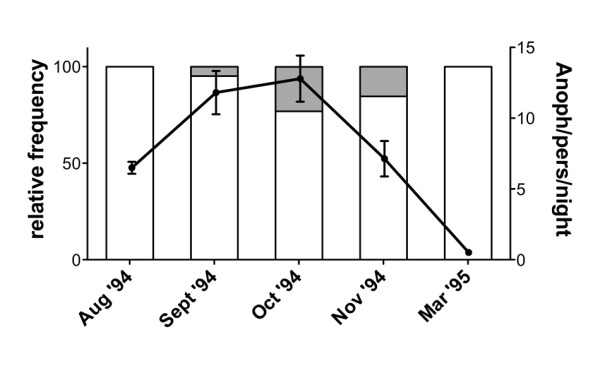
***Anopheles *density in the village of Barkoumbilen during the study period**. Total number of *Anopheles *females (*A. gambiae s.l*. and *A. funestus*) per person per night and their relative frequencies during the study period are reported. *A. gambiae *(empty area), *A. funestus *(grey area). Bars denote 95% confidence interval (CI). Data are based on pyrethrum spray catches carried out monthly (12 catches/month) as indicated. The total number of captured mosquitoes were as follows: Aug '94, n = 275; Sept '94, n = 547; Oct '94, n = 576; Nov '94, n = 235; Mar '95, n = 20.

To evaluate if human IgG directed against the *A. gambiae *gSG6 cross-reacted to the fSG6 protein and viceversa, the IgG response to both recombinant proteins was compared in 335 sera from individuals of the Mossi ethnic group collected in the village of Barkoumbilen (Burkina Faso) during three different surveys: at the beginning and the end of the high transmission/rainy season (Aug'94 and Oct'94) and in the following low transmission/dry season (Mar'95). In the study area the main malaria vectors were *A. gambiae, A. arabiensis *and *A. funestus*, with the members of the *A. gambiae *species complex (i.e. *A. gambiae *and *A. arabiensis*) being largely prevalent and representing, on average, approximately 90% of the indoor-resting *Anopheles *mosquitoes. *Anopheles funestus *reached a maximum toward the end of the high transmission season in Oct'94 (23.1%), which was also the period with the highest *Anopheles *density (12.6 *Anopheles*/person/night) as measured through the indoor pyrethrum spray catch method (Figure 2). The IgG response against recombinant gSG6 and fSG6 was compared by ELISA using sera from exposed individuals and European non-exposed controls. Considering the higher prevalence of *A. gambiae *in the study area, one would expect to find a differential response to the two antigens in case of low or moderate cross-reactivity; on the contrary, a similar pattern and a comparable intensity of the IgG response would be indicative of extensive cross-reactivity. Moreover, analysis of the response to a mixture of both gSG6 and fSG6 may provide indications concerning potential additive effects of their combined use.

### Seasonal and age-dependent pattern of the IgG response to gSG6 and fSG6

**Figure 3:**
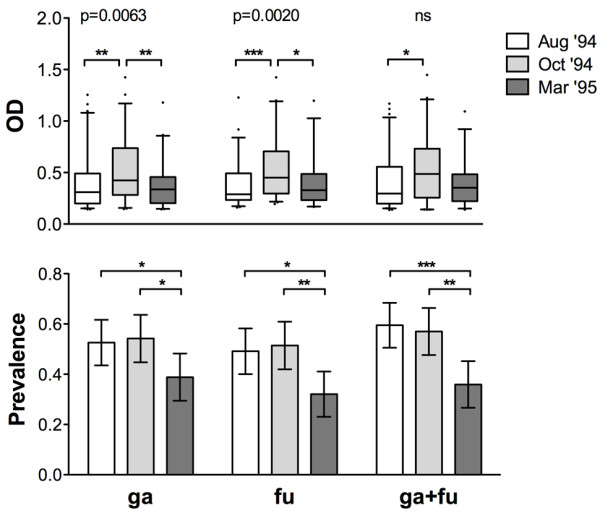
**Seasonal variation of the IgG response to gSG6, fSG6 or their combination**. IgG levels and prevalence to the different antigens: ga, gSG6; fu, fSG6; ga+fu, gSG6+fSG6. **(Top) **Box plot of OD values among responders to the indicated antigen in the different surveys. Box plots display the median OD value, 25^th ^and 75^th ^percentile. Whiskers represent 5-95 percentile and dots the outliers. P value was determined according to Kruskal-Wallis test. Pairwise comparisons refer to Mann-Whitney test (*, 0.01 < p < 0.05; **, 0.001 < p < 0.01; ***, p < 0.001). Number of responders (n) and mean age in years ± 95% CI (in parentheses) were as follows: Aug '94, [ga, n = 61 (15.8 ± 3.3); fu, n = 57 (17.5 ± 4.4); ga+fu, n = 69 (19.1 ± 4.3)]; Oct '94, [ga, n = 58 (16.5 ± 3.6); fu, n = 55 (17.0 ± 3.9); ga+fu, n = 61 (16.4 ± 3.5)]; Mar '95, [ga, n = 40 (15.0 ± 4.7); fu, n = 33 (14.9 ± 5.6); ga+fu, n = 37 (14.7 ± 4.9)]. **(Bottom) **Seasonal variation of seroprevalence to the different antigens in the three surveys. Whiskers denote the 95% CI. Number of individuals (n) and mean age as follows: Aug '94, n = 116 (20.2 ± 3.3); Oct '94, n = 107 (18.4 ± 2.8); Mar '95, n = 103 (17.8 ± 3.0). P values were determined by chi-square test (*, 0.01 < p < 0.05; **, 0.001 < p < 0.01).

As previously shown in the same epidemiological setting analysed here, the IgG response to the gSG6 antigen exhibits seasonal variations [20]. More specifically, both anti-gSG6 IgG levels and seroprevalence increased, or remained high, during the progression of the transmission/rainy season (Aug'94 to Oct'94) and dropped significantly during the following low transmission/dry season (Mar'95). The sub-sample analysed here showed, as expected, the same seasonal variation of the anti-gSG6 IgG response, and a very similar pattern was observed with the fSG6 protein or when a combination of the two antigens was used (Figure 3). More specifically, an increase of the OD levels among the responders was found for all antigens when comparing the start (Aug'94) to the end (Oct'94) of the high transmission season (Mann-Withney, gSG6: P = 0.006; fSG6: P = 0.0008; gSG6 + fSG6: P = 0.033). A significant decrease was observed during the following low transmission/dry period for gSG6 and fSG6 (Mann-Withney, Oct'94 vs. Mar'95, gSG6: P = 0.008; fSG6: P = 0.017), whereas the decrease was not significant with a mixture of the two antigens (Figure 3, upper panel). When seroprevalence was analysed, no significant changes were found during the high transmission/rainy season (Aug '94 to Oct '94), with values ranging from 49% to 59% depending on the antigen and the survey. On the contrary, a significant decrease to 32%-39% was observed during the following low transmission/dry period (chi-square, Aug'94 vs. Mar'95, gSG6: P = 0.042; fSG6: P = 0.010; gSG6 + fSG6: P = 0.0005. Oct'94 vs. Mar'95, gSG6: P = 0.026; fSG6: P = 0.004; gSG6 + fSG6: P = 0.002. Figure 3, lower panel). Therefore, overall, the analysis of the anti-SG6 IgG response, independently from the antigen used (gSG6, fSG6 or both), showed an overlapping pattern of seasonal variation in the exposed individuals.

**Figure 4:**
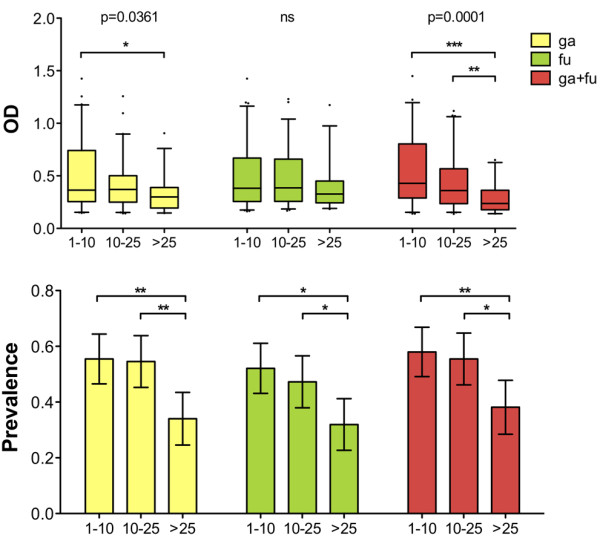
**Age variation of the IgG response to gSG6, fSG6 or their combination**. Age distribution of IgG levels and prevalence to the different antigens. Data from the three different surveys were pooled together. Age groups are indicated at the bottom. **(Top) **Box plot of OD values among responders to the different antigens. The number of responders (n) was as follows: ga (1-10, n = 66; 10-25, n = 60; >25, n = 33); fu (1-10, n = 62; 10-25, n = 52; >25, n = 31); ga+fu (1-10, n = 69; 10-25, n = 61; >25, n = 37). Box plots, whiskers, dots and p values as in Figure 3. **(Bottom) **Seroprevalence to the different antigens according to age. Number of individuals (n) was as follows: 1-10, n = 119; 10-25, n = 110; >25, n = 97. Whiskers and p values as in Figure 3.

Previous analysis also showed a peculiar age-dependent pattern of the anti-gSG6 IgG response in mosquito/malaria-exposed individuals from the two villages of Barkoundouba and Barkoumbilen, Burkina Faso [20]. Individuals from this area, as well as from all malaria endemic regions, show an age-related profile of increased seroprevalence and antibody levels against different *P. falciparum *antigens [22,23]; the humoral response appears low in children under five years of age and progressively increases to reach the highest level in adults. On the contrary, both the anti-gSG6 IgG levels and prevalence showed an age-dependent decrease, being higher in children up to ~10 years of age and progressively decreasing in adults [20]. A similar pattern of decrease with age was found here. In particular, independently from the antigen and the survey, the median OD value, 75% percentile, maximum OD value and prevalence were higher in 1-10 years old children as compared to >25 years-old individuals, although this difference reached statistical significance only in about half the cases (Additional file 1). The lack of statistical support, at least during the Mar '95 survey, is most likely the result of the seasonal decrease reported above and of the lower number of responders, which reduce sample size and tend to flatten age-related differences in the response during the dry season. A simplified overview of the anti-SG6 IgG response in the different age classes can be obtained if the three surveys are pooled together; this does not introduce a bias because individuals belonging to the different age classes are well partitioned in the three surveys (see Additional file 1). As shown in Figure 4 (upper panel) the anti-SG6 IgG levels were significantly lower in the sera of individuals older than 25 years when the gSG6 or both recombinant proteins were used as antigens, whereas the difference was not significant for the fSG6 protein. As far as the seroprevalence pattern is concerned, the frequency of responders was higher in children (1-10 years) and young/adults (10-25 years) than in older individuals (>25 years) although only in about half the cases this difference gets statistical support (Additional file 2). When data on the three surveys are pooled together, seroprevalence showed a very similar pattern for all the antigens tested and a significant decrease was observed in older individuals in comparison to the other two age classes, i.e 1-10 and 10-25 years-old (Figure 4, lower panel).

In summary, both the seasonal and the age-related pattern of the anti-SG6 humoral response were very similar with the gSG6 and the fSG6 recombinant proteins, or with their combination, and recapitulated what previously observed in the same epidemiological setting with the *A. gambiae *gSG6 antigen alone [20].

### Comparison of the IgG response to gSG6 and fSG6

**Figure 5:**
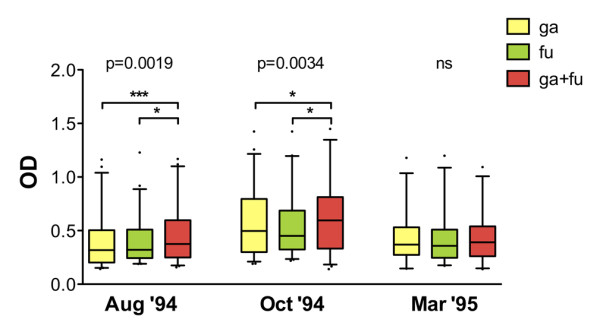
**Comparison of the IgG response to gSG6, fSG6 or their combination**. Box plot of OD values among responders to the three antigens in the different surveys as indicated. Box plots, whiskers and dots as in Figure 3. P value was determined according to Friedman test. Pairwise comparisons by the Wilcoxon matched-pairs signed rank test (two-tailed). Number of responders (n) and mean age in years ± 95% CI (in parentheses) were as follows: Aug '94, n = 46 (13.7 ± 3.7); Oct '94, n = 48 (15.9 ± 4.1); Mar '95, n = 28 (14.6 ± 6.3).

**Figure 6:**
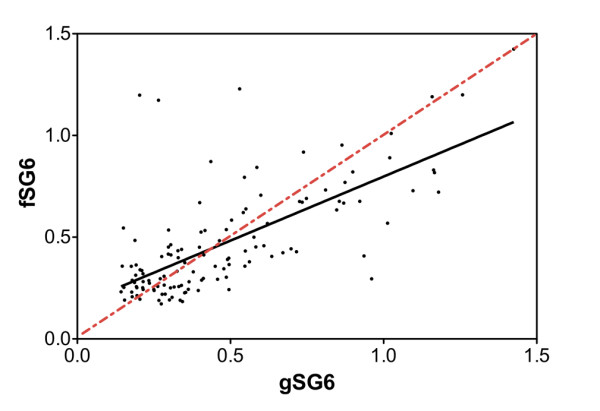
**Scatter plot analysis of IgG response to gSG6 and fSG6**. OD values among the 122 responders to both gSG6 and fSG6 in the three different surveys are reported. The best-fit is shown as a black line (slope 0.6301 ± 0.06137) and the diagonal as a red dotted line. Spearman correlation coefficient r = 0.6612, p < 0.0001.

Comparison of IgG levels and seroprevalence to gSG6, fSG6 or their combination in each of the three surveys did not show any significant difference (Kruskal-Wallis, Mann Whitney or chi-square tests) as also visible comparing the responses to the different antigens in Figure 3. When only responders to the three antigens in each survey were considered (Wilcoxon matched-pairs test), again no difference was found comparing the response to gSG6 and fSG6, however, a significant increase of IgG level was found during the high transmission/rainy season (Aug '94 and Oct '94) with a combination of both antigens (Figure 5). The absence of any difference during the dry season (Mar '95) may be ascribed, as mentioned earlier, to the lower IgG levels and the lower number of responders. These observations strongly support a wide cross-reactivity to the gSG6 and fSG6 salivary antigens and, in addition, suggest that the two proteins are most likely carrying species-specific epitopes, which would account for the increased anti-SG6 IgG level to their combination. According to this hypothesis, when the response to gSG6 and fSG6 was directly compared by scatter plot (Figure 6) some individuals showed higher response to the *A. funestus *protein (dots above diagonal) and others to the *A. gambiae *protein (dots below diagonal). Considering the higher prevalence of *A. gambiae s.l*. mosquitoes (always higher than 77%) it is not surprising that the best-fit line runs mostly below the diagonal. The simplest interpretation is that the higher response to fSG6 in some individuals is balanced by the higher response to gSG6 in others; this would give, as final result, the absence of any statistically significant difference between the responses to the two antigens. On the other hand, the simultaneous use of both recombinant proteins would imply an additive effect of the species-specific epitopes, which significantly increase the IgG levels in comparison to the individual antigens (Figure 5).

Overall, the analysis of the humoral response of exposed individuals from a malaria hyperendemic area from Burkina Faso to the recombinant SG6 protein from *A. gambiae *and *A. funestus *indicates that there is wide cross-reactivity to these two antigens. Both IgG level and seroprevalence obtained with either the gSG6 or the fSG6 antigens were very similar, even in an epidemiological setting where *A. gambiae *and *A. arabiensis*, both members of the *A. gambiae *complex, are largely prevalent (>77%). Since the SG6 proteins from these two last species are 99% identical, these observations support the use of the *A. gambiae *gSG6 as a reliable marker to evaluate human exposure to the three main Afrotropical malaria vectors: *A. gambiae*, *A. arabiensis *and *A. funestus*. In addition, this study provides evidence that gSG6 and fSG6 proteins also carry species-specific epitopes, suggesting that their combined use may allow for an increase in sensitivity, which may be especially relevant in conditions of low malaria transmission and/or low density of *Anopheles *vectors (dry season, post anti-vector control interventions).

### The gSG6 salivary protein: a marker of exposure to both Afrotropical and Asian malaria vectors?

**Figure 7:**
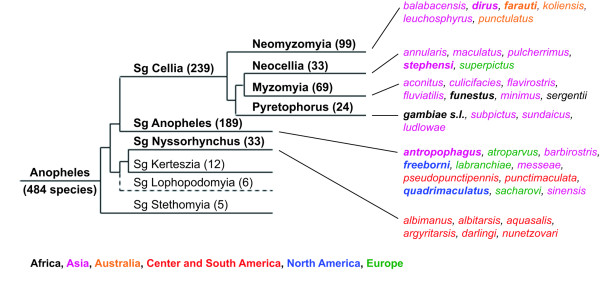
**Cladogram of relationships within the genus *Anopheles *with main malaria vectors**. The different subgenera constituting the genus *Anopheles *with number of species (in parentheses) are shown. A seventh subgenus, named *Baimaia *and represented by only one species [27] has been omitted. The three subgenera including the most relevant malaria vectors are in bold. Subgenus *Cellia *includes six Series but only the four relevant for malariology and including most of the species are shown. Primary malaria vectors belonging to the different Series/subgenus are shown on the right with color codes indicating their main geographic distribution. In bold are species for which partial or complete information on SG6 protein is available. Branch length do not indicate phylogenetic distance. Redrawn with permission from Besansky NJ *et al*. http://www.vectorbase.org/Help/Anopheles_species_cluster_white_paper.

Genus *Anopheles *includes ~500 species of which less than 40 play a dominant role in malaria transmission and are classified into one of three subgenera [26-28]. The subgenus *Cellia *includes all main African and Australian, as well as most of the Asian malaria vectors and can be further divided in six Series; the four ones which are relevant for malariology and include most of the species are schematically diagrammed in the cladogram in Figure 7. Most of the major Central and South American vectors belong to the subgenus *Nyssorhynchus *whereas the subgenus *Anopheles *includes mostly European, North American and a few additional Asian vectors.

SG6 family members are most likely absent in the subgenus *Nyssorhynchus *[18], whereas they have been found in the saliva of *A. freeborni *(*Anopheles *subgenus) and of important *Cellia *malaria vectors, namely *A. gambiae *and *A. arabiensis *(Pyretophorus), *A. funestus *(Myzomyia) and *A. stephensi *(Neocellia). Sequence comparison shows that the *A. freeborni *SG6 protein shares 61-62% identical amino acid residues with homologues from the *Cellia *subgenus which, in turn, are 80 to 84% identical among themselves (Table 1). The incomplete sequence of other members of the SG6 family from a few additional anopheline species can also be retrieved by searching ESTs (Expressed Sequence Tag) or SRA (Sequence Read Archive) databases: *Anopheles quadrimaculatus *(SRS008483, 65% identity to gSG6, 54/83 aa), *Anopheles anthropophagus *(FE969059, 64% identity to gSG6, 32/50 aa), *Anopheles dirus *(SRS008433, 68% identity to gSG6, 22/32 aa) and *Anopheles farauti *(SRS008445, 59% identity to gSG6, 13/22 aa). Therefore the SG6 protein is also present in the Neomyzomyia Series. It is noteworthy that the alignment of all the SG6 proteins identified so far shows a complete conservation of the 10 cystein residues, which indicates a very conserved folding, a feature certainly relevant for their recognition from the immune system.

**Table 1:** Comparison of SG6 proteins in selected malaria vectors

	*A. arabiensis*	*A. funestus*	*A. stephensi*	*A. freeborni*
*A. gambiae*	99% (86/87)	80% (70/87)	80% (70/87)	61% (53/87)
*A. arabiensis*		82% (71/87)	82% (71/87)	62% (54/87)
*A. funestus*			84% (73/87)	61% (53/87)
*A. stephensi*				62% (54/87)

Percentage of identical amino acid residues among SG6 proteins from *Anopheles gambiae*, *A. arabiensis*, *A. funestus*, *A. stephensi *and *A. freeborni *is reported. The number of identical/total amino acid residues is shown in parentheses.

The wide cross-reactivity between the *A. gambiae *and *A. funestus *SG6 proteins, along with the observations reported above, supports the idea that the anti-gSG6 IgG response may represent a reliable indicator of exposure to malaria vectors of the subgenus *Cellia*, at least to members of the Pyretophorus, Myzomyia and Neocellia Series, which share 80% to 84% identity. Therefore, it is likely that the gSG6 protein will also work as marker of exposure to *A. stephensi *and other Asian malaria vectors. Furthermore, the highly conserved folding and the relatively high identity (61-65%) indicates that anti-gSG6 antibodies are also expected to cross-react with SG6 family members from species of the *Anopheles *subgenus, although extension of cross-reaction and its potential usefulness would need proper validation.

## Conclusions

This study provided solid experimental evidence that the human antibody response to the *A. gambiae *salivary protein gSG6 represents a reliable indicator of human exposure to the three main malaria vectors in tropical Africa: *A. gambiae*, *A. arabiensis *and *A. funestus*. Such a tool may be very useful for malaria epidemiological studies and for monitoring vector control interventions. The additive effect obtained when the two recombinant proteins are combined also allows for an increase in sensitivity of the assay. Moreover, data reported here also suggest that most likely the gSG6 protein may work as a marker of exposure to *A. stephensi *and other Asian malaria vectors or, as alternative, that inclusion of SG6 protein from a third species may provide a sensitive marker of human exposure to bites of both African and Asian malaria vectors.

## Competing interests

The authors declare that they have no competing interests.

## Authors' contributions

CR performed the immunoassays and contributed to data analysis. RR carried out expression and purification of recombinant proteins. GF participated in the design of the study and in setting conditions for protein purification. VM participated in the design of the study and analysis of data. SB and NI contributed reagents. VP contributed to study design and to data and statistical analyses. DM conceived the study, participated to its design and analysis of data, contributed reagents. BA conceived and coordinated the study, participated to data and statistical analyses, wrote the manuscript. All authors read and approved the final manuscript.

## Supplementary Material

Additional file 1 **IgG response to the different antigens in the three surveys according to age**. Box plots of OD values among responders to the indicated antigen (ga, gSG6; fu, fSG6; ga+fu, gSG6+fSG6) in the three different surveys. Box plots display the median OD value, 25^th ^and 75^th ^percentile. Whiskers represent 5-95 percentile and dots the outliers. P values refer to pairwise comparisons according to Mann-Whitney test. Age classes are indicated at the bottom and the number of responders is in parentheses. Click here for file

Additional file 2 **Seroprevalence to the different antigens in the three surveys according to age**. Seroprevalences to the different antigens in the three surveys are indicated in the legends. Age classes are at the bottom and the number of responders on total individuals is shown in parentheses. Whiskers denote the 95% CI. P values were determined by the chi-square test. Click here for file
