# Supplementary figures and images for: Wide cross-reactivity between Anopheles gambiae and Anopheles funestus SG6 salivary proteins supports exploitation of gSG6 as a marker of human exposure to major malaria vectors in tropical Africa
Source: Malar J. 2011 Jul 27;10:206. doi: 10.1186/1475-2875-10-206 (PMC3160432; doi:10.1186/1475-2875-10-206)

Aug '94 ga

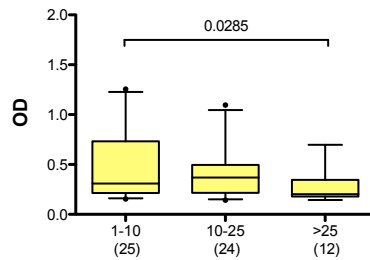

Aug '94 fu

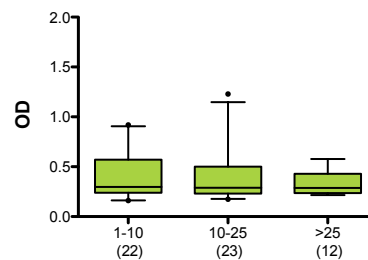

Aug '94 ga+fu

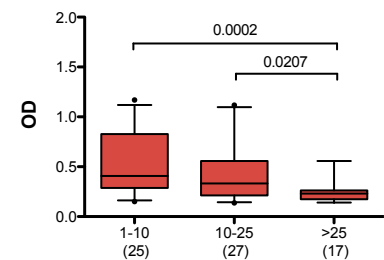

Oct '94 ga

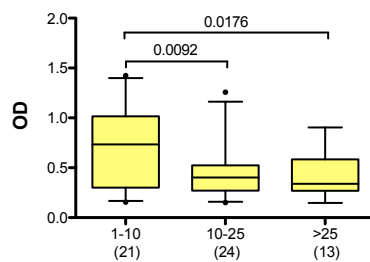

Oct '94 fu

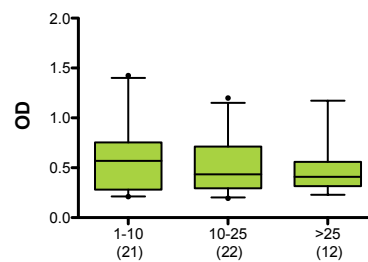

Oct '94 ga+fu

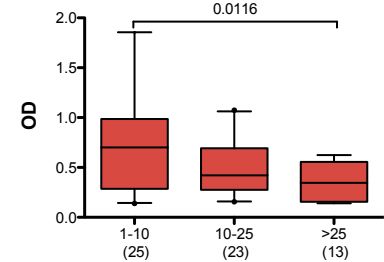

Mar '95 ga

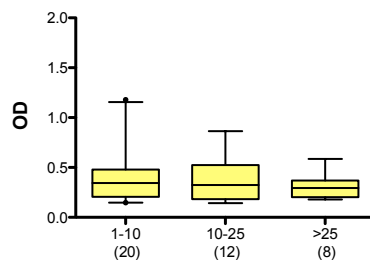

Mar '95 fu

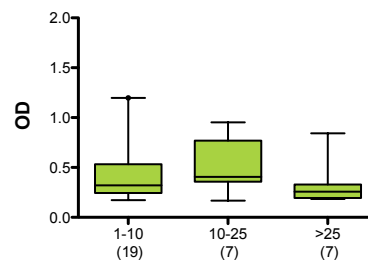

Mar '95 ga+fu

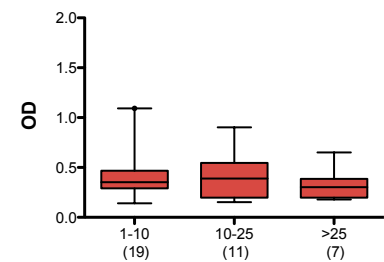

Supplement: Additional file 1 — IgG response to the different antigens in the three surveys according to age. Box plots of OD values among responders to the indicated antigen (ga, gSG6; fu, fSG6; ga+fu, gSG6+fSG6) in the three different surveys. Box plots display the median OD value, 25th and 75th percentile. Whiskers represent 5-95 percentile and dots the outliers. P values refer to pairwise comparisons according to Mann-Whitney test. Age classes are indicated at the bottom and the number of responders is in parentheses. [file 1475-2875-10-206-S1.PDF]

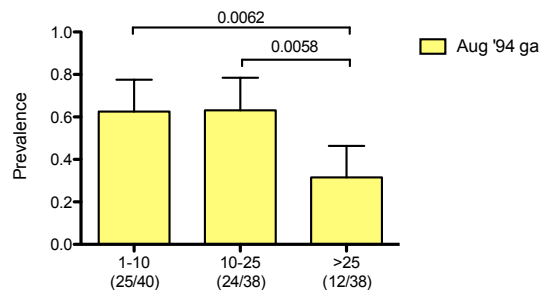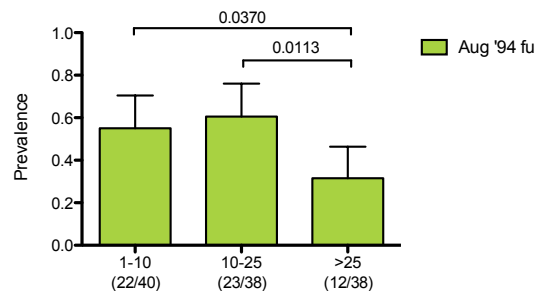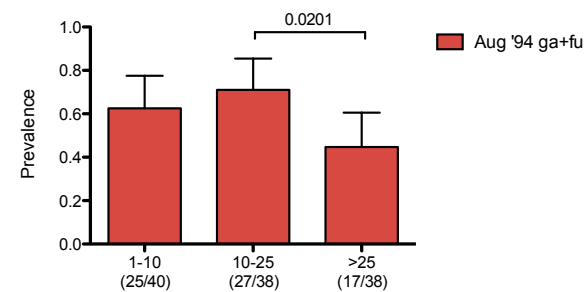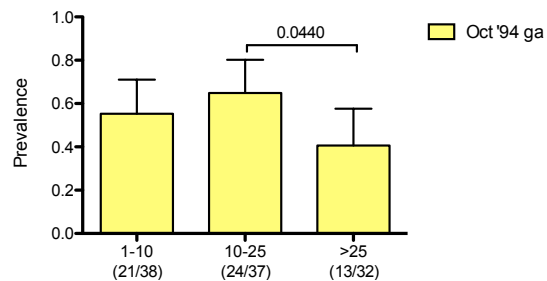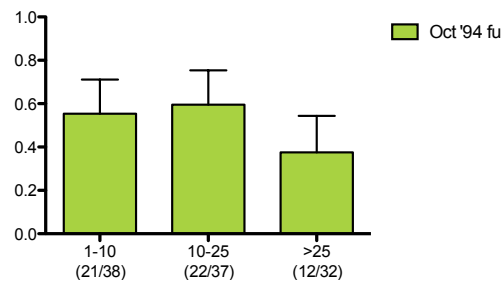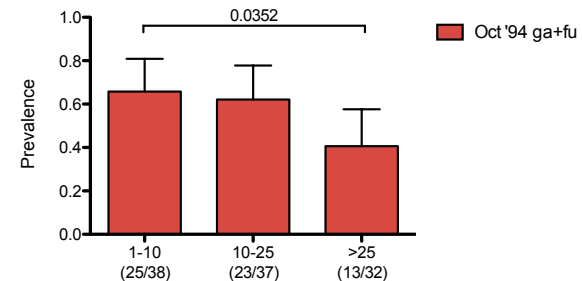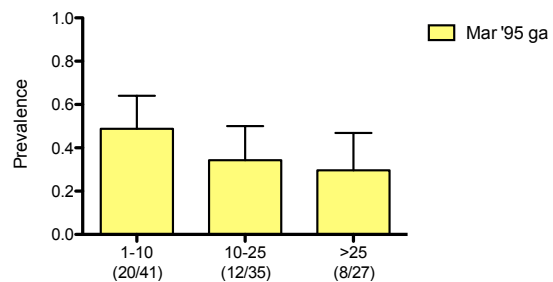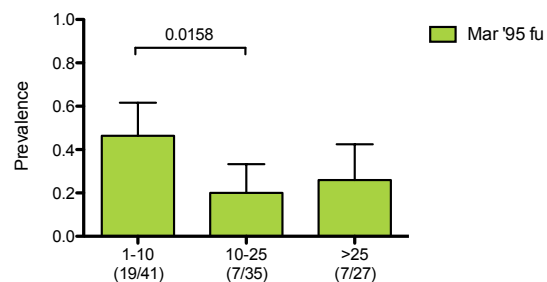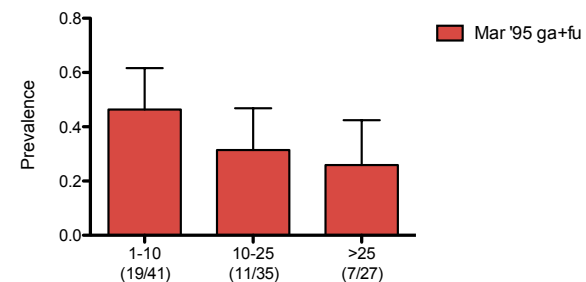

Supplement: Additional file 2 — Seroprevalence to the different antigens in the three surveys according to age. Seroprevalences to the different antigens in the three surveys are indicated in the legends. Age classes are at the bottom and the number of responders on total individuals is shown in parentheses. Whiskers denote the 95% CI. P values were determined by the chi-square test. [file 1475-2875-10-206-S2.PDF]
